# The methylenetetrahydrofolate reductase (MTHFR) C677T gene polymorphism is associated with breast cancer subtype susceptibility in southwestern China

**DOI:** 10.1371/journal.pone.0254267

**Published:** 2021-07-09

**Authors:** Zhen Li, Ji Zhang, Wei Zou, Qi Xu, Siyuan Li, Jie Wu, Li Zhu, Yunjiao Zhang, Lei Xu, Ying Zhang, Qingsong Luo, Jianyun Nie, Xingxu Li, Tianning Zou, Ceshi Chen

**Affiliations:** 1 Department of Breast Surgery, Yunnan Tumor Hospital, The Third Affiliated Hospital of Kunming Medical University, Kunming, Yunnan, China; 2 Queen Mary Institute, Nanchang University, Nanchang, China; 3 Department of Molecular Biosciences, Institute of Cellular and Molecular Biology, The University of Texas, Austin, Texas, United States of America; 4 Key Laboratory of Animal Models and Human Disease Mechanisms of Chinese Academy of Sciences & Yunnan Province, Kunming Institute of Zoology, Kunming, Yunnan, China; 5 Yunnan Economy & Society Bigdata Research Institute, Yunnan University of Finance and Economics, Kunming, Yunan, China; 6 Kunming Medical University Haiyuan College, Kunming, Yunnan, China; 7 China Affiliated Cancer Hospital & Institute of Guangzhou Medical University, Guangzhou, China; Fondazione IRCCS Istituto Nazionale dei Tumori, ITALY

## Abstract

Methylenetetrahydrofolate reductase (MTHFR), a folate-dependent enzyme, is reportedly involved in several cancer types. The *MTHFR* C677T polymorphism influences many biological processes, including tumorigenesis. However, the association between the *MTHFR* C677T polymorphism and breast cancer (BC) subtypes is not fully understood. In this study, the *MTHFR* C677T polymorphism was genotyped in 490 individuals with or without BC from southwestern China. Analysis of the association between the *MTHFR* C677T polymorphism and BC revealed that there was a significant association between the *MTHFR* C677T polymorphism and triple-negative breast cancer (TNBC) (OR = 2.83, 95% CI: 1.12–9.51, *P* = 0.0401). Furthermore, the *MTHFR* C677T polymorphism can also serve as a protective factor in luminal A breast cancer (OR = 0.57, 95% CI: 0.34–0.94, *P* = 0.0258). Evaluation of the association between the *MTHFR* C677T polymorphism and clinical characteristics indicated that people who suffered from hypertension had an increased risk for BC (OR = 2.27; 95% CI: 1.08–4.6; *P* = 0.0264), especially TNBC (OR = 215.38; 95% CI: 2.45–84430.3; *P* = 0.0317). Our results suggest that the *MTHFR* C677T polymorphism is significantly associated with susceptibility to luminal B breast cancer and TNBC.

## Introduction

Breast cancer (BC) is the most common type of cancer and the leading cause of cancer-related death in women worldwide [1]. More than 90% of breast cancers are not metastatic at the time of diagnosis. For people presenting without metastatic disease, therapeutic goals are tumor eradication and preventing recurrence [2]. Breast cancer is categorized into 4 major subtypes based on unique genetic alterations and the presence or absence of molecular markers for estrogen or progesterone receptors and human epidermal growth factor 2 (Her 2): luminal A, luminal B, Her2^+^ and triple negative breast cancer (TNBC) [3]. Systemic therapy for both nonmetastatic and metastatic breast cancer is determined by subtype, with the goals of prolonging life and palliating symptoms. As a result, the term “BC” no longer refers to a single disease but rather a heterogeneous group of diseases associated with a diversity of tumor backgrounds/subtypes.

However, clinically, breast cancer is more aggressive and is associated with a poorer prognosis in younger women than in older women, especially in TNBC patients [4]. Targeted therapies for germline mutations in BRCA1 or BRCA2 and PI3KCA have demonstrated clinical efficacy in breast cancers [5, 6]. In this respect, many ongoing studies aim to screen biomarkers as potential predictors of prognosis or response to therapy, which will most likely lead to individualized management of the disease.

Folate (FA), a genetic term for a B-group vitamin (vitamin B9), is important for cell division and homeostasis due to its essential role in the synthesis of S-adenosyl-methionine, the methyl donor required for all methylation reactions in cells [7]. As an important 1-carbon source, folate is involved in many cellular processes, including DNA synthesis, maintenance and epigenetics. Low folate status or insufficient intake has been associated with an increased risk of neural tube defects, cancers and cardiovascular disease [8, 9]. Chen et al. demonstrated that folate causes normal cells to turn cancerous [10], and dietary folate intake was associated with DNA methylation in early-stage breast cancer [11]. In addition, Friso et al [12] found that reduced global DNA methylation and lower plasma folate concentration were associated with genotype-dependent methylenetetrahydrofolate reductase (MTHFR).

MTHFR is a folate-dependent enzyme that catalyzes and converts 5,10-methylenetetrahydrofolate to 5-methyltetrahydrofolate. The *MTHFR* gene is involved in many biological processes, including the regulation of intracellular folate levels, DNA synthesis, methylation and tumorigenesis [13]. There are two well-studied polymorphisms of the *MTHFR* gene, *MTHFR* 677C> T and 1298A> C. Large studies have indicated that the *MTHFR* 677C> T polymorphism may play a role in the development of breast cancer [14, 15]. Despite the existence of a variety of data for studies on the association between these polymorphisms and breast cancer, findings of genetic association have been inconsistent for a large body of conditions.

Furthermore, previous epidemiological studies indicated C677T and A1298C *MTHFR* polymorphisms were related to breast cancer susceptibility in Latinos [16]. However, these candidate *MTHFR*-associated loci have not been investigated in southwestern Chinese populations. Accordingly, the present study was designed to replicate the reported gene SNPs, aiming to identify *MTHFR* polymorphisms and their correlation with susceptibility to different subtypes of breast cancer. As a result, we found that *MTHFR* 677C> T exhibited differential expression in breast cancer. Therefore, *MTHFR* rs1801133 may serve as a novel target indicating lower risk in different subtypes of breast cancer in southwestern Chinese patients.

## Patients and methods

### Study subjects

All subjects were from southwestern China and were collected randomly at Yunnan Tumor Hospital in China. Patients were recruited from January 2012 to December 2017 in Yunnan Tumor Hospital and had been diagnosed with BC. Some patients were excluded due to a lack of gene sequencing. This case-control study included 490 BC patients and 490 cancer-free controls. Subtypes luminal A, luminal B, Her2^+^ and TNBC were classified by histochemistry. For patient recruitment, controls without a history of cancer were selected based on physical examination in the same region during the same period. Age and sex were used for frequency matching to cases and demographic information, and the clinical characteristics of each participant were collected at the same time.

### Genotyping

DNA was extracted from leukocytes using a Wizard Genomic DNA Purification Kit (Promega Corporation, FL, USA) according to the manufacturer’s instructions. Genotyping of the *MTHFR* C677T polymorphism (rs1801133) was performed using matrix-assisted laser desorption/ionization time-of-flight mass spectrometry. Primers for amplification of MTHFR 677C>T were (forward) 5′- TGA CCT GAA GCA CTT GAA GGA GAA -3′ and (reverse) 5′- GGA AGA ATG TGT CAG CCT CAA AGA -3′.

## Statistical analysis

The association of the *MTHFR* C677T polymorphism with BC incidence was calculated using adjusted odds ratios (AORs) and 95% confidence intervals (CIs) by multivariate logistic regression. *Χ*^*2*^ tests were used to examine the deviation of genotype frequency in Hardy-Weinberg equilibrium (HWE) and to analyze differences in genotype distribution, clinicopathological features and physicochemical characteristics using the *R* program (Version 3.61, USA). Overall survival time was calculated from the date of cancer diagnosis to the date of death. A *P*-value less than 0.05 was considered statistically significant.

## Ethics statement

Our study protocol was approved by the Institutional Review Board of Kunming Medical University. All study subjects provided written informed consent to participate in the study. This study conformed to the tenets of the Declaration of Helsinki. All methods applied were performed in accordance with the approved guidelines.

## Results

### Cases and controls

We recruited a total of 490 BC cases and cancer-free controls. The genotype frequency of *MTHFR* C677T in the controls and cases was in concordance with Hardy-Weinberg equilibrium (HWE) (*P* = 0.69 and *P* = 0.45, respectively). The demographic characteristics of the cases and controls are presented in Table 1.

**Table 1:** Baseline characteristics of breast cancer patients and control subjects.

Characteristics	BC	Control	*p*
Number	490	490	
Age: years (mean± SDc)	47.9± 9.8	49.9± 16.1	0.46
Diabetes Mellitus: n%	22 (4.5%)	14 (2.9%)	0.234
Hypertension: n%	51 (10.4%)	12 (2.4%)	< 0.001
Menopause: n%	178 (36.3%)	192 (39.2%)	0.392
BMI≥ 25 kg/m2: n%	149 (30.4%)	127 (25.9%)	0.136
Cholesterol> 5.17 mmol/L: n%	175 (35.7%)	94 (19.2%)	< 0.05
Triglyceride> 1.7 mmol/L: n%	173 (35.3%)	157 (32%)	0.311
Family History	44 (9.0%)		
Tumor site: n%			
Left	246(50.2%)		
Right	234(47.8%)		
Bilateral	9 (1.8%)		
Tumor size: n%			
< 5 cm	368 (75.1%)		
≥ 5 cm	69 (14.1%)		
Lymph node Metastasis: n%			
1≤N≤ 3	108 (22.0%)		
4≤N≤ 9	53 (10.8%)		
N> 9	23 (4.7%)		
N0	305 (62.2%)		
Distance Metastasis: n%	44 (9.0%)		
TNM stage: n%			
I+II	313 (63.9%)		
III+IV	131 (26.7%)		

Abbreviations: *BC*, breast cancer; *SD*, standard deviation; *BMI*, body mass index; *LNM*, lymph node metastasis; *MTHFR*, methylenetetrahydrofolate reductase; *TNM*, tumor node metastasis. *P*-values were calculated using *Χ*^2^ test for categorical data and two-sided t-test for continuous data.

In this case-control study, the distribution of age was consistently investigated among all cases and controls (*P* = 0.46). The allelic frequency of *MTHFR* 677T was 0.44 in the controls and 0.47 in the BC cases. Considering that BC is a complicated disease, many risk factors, such as body weight and menopause, are associated with the occurrence and progression of BC [17]. Therefore, we conducted a stratified analysis on data according to age, diabetes mellitus, presence of hypertension, family history of cancer, menopause, body mass index (BMI), tumor site, tumor size, and lymph node metastasis (LNM) stage to determine whether the *MTHFR* C677T polymorphism is associated with BC incidence in specific subtypes of the study population, as shown in Table 1.

### The *MTHFR* C677T polymorphism and BC

Considering the small number of *MTHFR* AA genotypes, the dominant genetic model (CC/CT+TT) was used to analyze the genotype distribution of the *MTHFR* C677T polymorphism. For all BCs, there was a significant association with the *MTHFR* C677T polymorphism (OR = 1.55, 95% CI: 1.12–2.16, *P* = 0.0082) in the dominant genetic model. According to IHC classification, consistent with the results for all BCs, there were significant associations between the *MTHFR* C677T polymorphism and luminal A (OR = 0.57, 95% CI: 0.34–0.94, *P* = 0.0258) and TNBC (OR = 2.83, 95% CI: 1.12–9.51, *P* = 0.0401) disease subtypes. No significant association was observed between the *MTHFR* C677T polymorphism and luminal B (OR = 1.19, 95% CI: 0.75–1.94, *P* = 0.4661) or Her2+ (OR = 0.96, 95% CI: 0.56–1.73, *P* = 0.8856). For estrogen and progesterone status, no association was observed between the *MTHFR* C677T polymorphism and ER+ (OR = 0.92, 95% CI: 0.63–1.34, *P* = 0.6537), ER− (OR = 1.30, 95% CI: 0.77–2.28, *P* = 0.3479), PR− (OR = 1.54, 95% CI: 0.89–2.82, *P* = 0.1419) or Her2+ (OR = 0.96, 95% CI: 0.56–1.73, *P* = 0.8856), as shown in Table 2.

**Table 2:** Genotype distribution of the *MTHFR* C677T polymorphism in controls and patients by BC subtype.

Category	CC		CT+TT		OR (95%CI)	*p*
	n	%	n	%		
Control (490)	110	22.4	377	76.9	1.00	
All BC (490)	76	15.5	413	84.3	1.55 (1.12–2.16)	**0.0082**
IHC subtype						
Luminal A (99)	24	4.9	75	15.3	0.57 (0.34–0.94)	**0.0258**
Luminal B (205)	28	5.7	177	36.1	1.19 (0.75–1.94)	0.4661
Her2^+^ (112)	18	3.7	93	19.0	0.96 (0.56–1.73)	0.8856
TNBC (65)	4	0.8	61	12.4	2.83 (1.12–9.51)	**0.0401**
ER and PR status						
ER+ (357)	61	12.4	296	60.4	0.92 (0.63–1.34)	0.6537
ER- (126)	13	2.7	112	22.9	1.30 (0.77–2.28)	0.3479
PR- (121)	11	2.2	109	22.2	1.54 (0.89–2.82)	0.1419
Her2^+^ (112)	18	3.7	93	19.0	0.96 (0.56–1.73)	0.8856
Tumor site						
Left	34	6.9	208	42.4	0.88(0.58–1.32)	0.4987
Right	36	7.3	194	39.6	1.24(0.79–1.98)	0.3548
Bilateral	2	0.4	7	1.4	0.66(0.16–4.50)	0.6103

### The *MTHFR* C677T polymorphism and clinical risk of BC

To understand the association between the *MTHFR* C677T polymorphism and the clinical features of the 4 subtypes of BCs, logistic regression analysis was used to evaluate the association between the *MTHFR* C677T polymorphism and clinical characteristics (age at diagnosis and menarche, diabetes mellitus, presence of hypertension, cancer family history, menopause, BMI, tumor size, LNM stage, distant metastasis and tumor stage classification), as shown in Table 3.

**Table 3:** Clinicopathological features of BC patients classified by tumor site and *MTHFR* C677T polymorphism. The total number of individuals may not be the same due to censored data.

Variables	All BCs				luminal A				luminal B				HER2^+^				TNBC			
	CC	CT+TT	OR (95%CI)	*P*	CC	CT+TT	OR (95%CI)	*P*	CC	CT+TT	OR (95%CI)	*P*	CC	CT+TT	OR (95%CI)	*P*	CC	CT+TT	OR (95%CI)	*P*
Age (years)																				
>45 (1)	46	222	1.00	0.6645	15	44	1.00	0.5197	17	87	1.00	0.2128	13	52	1.00	0.9209	1	35	1.00	0.6679
≤45 (0)	30	191	1.19 (0.54–2.59)		10	31	0.64 (0.16–2.46)		10	90	2.26 (0.63–8.34)		5	41	1.10 (0.18–6.76)		3	26	2.33 (0.04–141.26)	
Age at menarche (years)																				
>15(1)	16	80	1.00	0.8534	5	12	1.00	0.7035	6	30	1.00	0.5496	5	28	1.00	0.5108	0	10	1.00	0.9958
≤15(0)	60	332	1.06 (0.56–1.91)		20	63	1.25 (0.36–3.87)		21	146	1.35 (0.46–3.48)		13	65	0.67 (0.19–2.09)		4	51	0 (Inf-1.53e+140)	
Diabetes Mellitus																				
Yes	5	17	1.00	0.4421	1	7	1.00	0.3205	4	4	1.00	**0.0092**	0	2	1.00	0.9928	0	4	1.00	0.9961
No	71	396	1.51 (0.48–4.02)		24	68	0.33 (0.02–2.1)		23	173	7.22 (1.56–33.64)		18	91	0 (Inf-3.95e+108)		4	57	0 (Inf-4.36e+155)	
Hypertension																				
Yes	14	36	1.00	**0.0264**	4	9	1.00	0.7535	6	15	1.00	0.0557	3	8	1.00	0.6043	1	4	1.00	**0.0317**
No	62	376	2.27 (1.08–4.6)		21	66	1.24 (0.3–4.45)		21	162	3.17 (0.93–10.23)		15	84	1.50 (0.28–6.46)		3	57	215.38 (2.45–84430.3)	
Family History of Cancer																				
Yes	6	38	1.00	0.7332	1	7	1.00	0.4297	1	11	1.00	0.6206	3	11	1.00	0.5338	1	8	1.00	0.5978
No	70	375	0.86 (0.32–1.96)		24	68	0.42 (0.02–2.56)		26	166	0.59 (0.03–3.24)		15	82	1.56 (0.32–5.88)		3	53	1.96 (0.08–20.15)	
Menopause																				
Yes	28	151	1.00	0.3154	8	26	1.00	0.2596	9	54	1.00	0.7811	11	37	1.00	0.4453	0	31	1.00	0.9955
No	48	262	0.69 (0.33–1.41)		17	49	0.45 (0.1–1.75)		18	123	0.84 (0.24–2.74)		7	56	1.77 (0.42–8.08)		4	30	0 (Inf-2.86e+156)	
Body Mass Index (kg/m^2^)																				
>25	29	117	1.00	0.1098	10	19	1.00	0.1900	14	52	1.00	**0.0289**	4	26	1.00	0.621	1	17	1.00	0.9932
≤25	47	295	1.52 (0.9–2.52)		15	56	1.90 (0.71–4.96)		13	124	2.51 (1.1–5.79)		14	67	0.74 (0.19–2.31)		3	44	0.99 (0.04–9.44)	
Tumor Size (cm)																				
>5	10	51	1.00	0.7988	2	7	1.00	0.8059	5	20	1.00	0.2547	3	19	1.00	0.9587	0	3	1.00	0.9964
≤5	58	309	1.10 (0.5–2.23)		22	61	0.81 (0.11–3.7)		18	133	1.90 (0.57–5.44)		13	65	1.04 (0.21–4)		4	47	0 (Inf-3.1e+195)	
Lymph Node Metastasis																				
Yes	32	155	1.00	0.4008	12	29	1.00	0.3573	8	66	1.00	0.5118	10	30	1.00	0.0567	1	30	1.00	0.5268
No	44	257	1.24 (0.75–2.04)		13	46	1.54 (0.61–3.92)		19	111	0.74 (0.29–1.76)		8	62	2.77 (0.98–8.16)		3	31	0.46 (0.02–4.27)	
Distance Metastasis																				
Yes	6	38	1.00	0.7127	2	3	1.00	0.4842	2	16	1.00	0.8024	2	11	1.00	0.9586	0	7	1.00	0.9948
No	70	375	0.84 (0.31–1.94)		23	72	1.95 (0.24–12.65)		25	161	0.82 (0.13–3.14)		16	82	0.96 (0.14–4.09)		4	54	0 (Inf-8.81e+103)	
Tumor Stage																				
III+IV(1)	20	112	1.00	0.8331	7	18	1.00	0.8153	6	48	1.00	0.7043	6	27	1.00	0.8354	0	17	1.00	0.9963
I+II(0)	50	262	0.94 (0.53–1.63)		18	52	1.13 (0.39–3.08)		17	114	0.83 (0.28–2.13)		12	56	1.12 (0.35–3.3)		3	39	0 (Inf-1.35e+196)	

In all BCs, the *MTHFR* (rs1801133) CT and TT genotypes were associated with elevated blood pressure (OR = 2.27; 95% CI: 1.08–4.6; *P* = 0.0264). Among the 4 BC subtypes, notably, in luminal B breast cancer, *MTHFR* CT and TT genotypes showed a strong association with diabetes mellitus (OR = 7.22; 95% CI: 1.56–33.64; *P* = 0.0092) and a higher BMI (OR = 2.51; 95% CI: 1.1–5.79; *P* = 0.0289). In addition, for TNBC, the *MTHFR677T* allele was associated with a very high risk of hypertension (OR = 215.38; 95% CI: 2.45–84430.3; *P* = 0.0317). There were no significant associations between the *MTHFR* C677T polymorphism and age at diagnosis or menarche, cancer family history, menopause, tumor size, LNM stage, distant metastasis or tumor stage classification. In addition, the *MTHFR* C677T polymorphism was not associated with other clinical features of BC in our study.

### The *MTHFR* C677T polymorphism and physicochemical characteristics of BC

The *MTHFR* gene is involved in many biological processes, including the regulation of intracellular folate levels. Therefore, we analyzed the association of the *MTHFR* C677T polymorphism with inflammatory, physiological, and biochemical indices in BC patients. The levels of neutrophils, leukocytes, cholesterol, hemoglobin, platelets, albumin, ASTa, ASTb, glucose, cholesterol, triglycerides, high-density lipoprotein cholesterol (HDL-c), and low-density lipoprotein cholesterol (LDL-c) were used to assess the association with the *MTHFR* C677T polymorphism in BC patients (Table 4). In all BC patients, GA and AA genotypes were more frequent in patients with glucose content exceeding 6.11 than in those with lower glucose content (OR = 2.52; 95% CI: 1.23–4.95; *P* = 0.0091). However, the association between the *MTHFR* C677T polymorphism and glucose content disappeared in patients with luminal A, Her2^+^ or TNBC but appeared to have a strong association with luminal B (OR = 4.37; 95% CI: 1.32–13.54; *P* = 0.0076). Except for glucose content, other clinical features, as listed in Table 4, were not associated with the *MTHFR* C677T polymorphism.

**Table 4:** Physicochemical characteristics of BC patients classified by tumor site and *MTHFR* C677T polymorphism. The total number of individuals may not be the same due to censored data.

Variables	All BCs				luminal A				luminal B				HER2^+^				TNBC			
	CC	CT +TT	OR (95%CI)	*P*	CC	CT +TT	OR (95%CI)	*P*	CC	CT +TT	OR (95%CI)	*P*	CC	CT +TT	OR (95%CI)	*P*	CC	CT +TT	OR (95%CI)	*P*
Neutrophil (%)																				
≤75	67	357	1.00	0.8542	19	66	1.00	0.3176	24	150	1.00	1.0000	18	82	1.00	0.2061	4	53	1.00	1.0000
>75	9	56	1.17 (0.54–2.81)		5	9	0.52 (0.14–2.23)		4	27	1.08 (0.33–4.62)		0	11	Inf (0.50-Inf)		0	8	Inf (0.09-Inf)	
Albumin (g/L)																				
<40	8	41	1.00	0.8366	4	4	1.00	0.0945	1	18	1.00	0.4812	2	7	1.00	0.6372	1	12	1.00	1.0000
≥40	68	372	1.07 (0.41–2.44)		20	71	3.49 (0.60–20.58)		27	159	0.33 (0.008–2.26)		16	86	1.53 (0.14–9.08)		3	49	1.35 (0.02–18.67)	
AST^a^ (U/L)																				
>35	8	32	1.00	0.4925	3	5	1.00	0.3971	2	16	1.00	1.0000	3	7	1.00	0.2050	0	4	1.00	1.0000
≤35	68	381	1.40 (0.53–3.27)		21	70	1.98 (0.28–11.21)		26	161	0.77 (0.08–3.62)		15	86	2.43 (0.37–12.24)		4	57	0 (0–29.18)	
ALT^b^ (U/L)																				
≥40	8	36	1.00	0.6618	5	5	1.00	0.0592	1	17	1.00	0.4775	2	8	1.00	0.6641	0	6	1.00	1.0000
<40	68	377	1.23 (0.47–2.85)		19	70	3.62 (0.75–17.58)		27	160	0.35 (0.008–2.42)		16	85	1.32 (0.13–7.55)		4	55	0 (0–16.89)	
Glucose (mmol/L)																				
>6.11	16	40	1.00	**0.0091**	4	9	1.00	0.5103	7	13	1.00	**0.0076**	4	6	1.00	0.0550	1	10	1.00	0.5391
≤6.11	59	372	2.52 (1.23–4.95)		20	66	1.46 (0.30–5.96)		20	164	4.37 (1.32–13.54)		14	87	4.07 (0.75–19.82)		3	50	1.65 (0.03–23.14)	
Cholesterol (mmol/L)																				
>5.17	28	147	1.00	0.8971	6	26	1.00	0.4559	12	64	1.00	0.5312	8	37	1.00	0.7963	1	18	1.00	1.0000
≤5.17	48	261	1.04 (0.60–1.77)		18	48	0.62 (0.18–1.89)		16	113	1.32 (0.53–3.19)		10	55	1.19 (0.37–3.70)		3	41	0.76 (0.01–10.26)	
Triglyceride (mmol/L)																				
>1.7	30	143	1.00	0.5148	8	17	1.00	0.4188	13	67	1.00	0.4099	8	34	1.00	0.6011	1	23	1.00	1.0000
≤1.7	46	265	1.21 (0.70–2.05)		16	57	1.67 (0.52–5.05)		15	110	1.42 (0.58–3.42)		10	58	1.36 (0.42–4.26)		3	36	0.53 (0.01–7.02)	
HDL-c^c^ (mmol/L)																				
<1.29	33	218	1.00	0.1333	10	35	1.00	0.6470	11	98	1.00	0.1532	8	51	1.00	0.4453	2	32	1.00	1.0000
≥1.29	43	190	0.67 (0.39–1.13)		14	39	0.80 (0.28–2.22)		17	79	0.52 (0.21–1.26)		10	41	0.65 (0.20–2.01)		2	27	0.85 (0.06–12.40)	
LDL-c^d^(mmol/L)																				
<3.36	56	293	1.00	0.7823	20	57	1.00	0.5819	22	131	1.00	0.8154	10	59	1.00	0.5957	3	43	1.00	1.0000
≥3.36	20	115	1.10 (0.62–2.02)		4	17	1.49 (0.41–6.79)		6	46	1.29 (0.47–4.12)		8	33	0.70 (0.22–2.26)		1	16	1.11 (0.08–62.20)	

Abbreviations:

^a^, glutamic oxalacetic transaminase;

^b^, glutamic-pyruvic transaminase;

^c^, high-density lipoprotein cholesterol;

^d^, low-density lipoprotein cholesterol;

* The *P* value shown in Table did not make an adjustment by clinical characteristics.

### The *MTHFR* C677T polymorphism and BC patient survival

**Fig 1:**
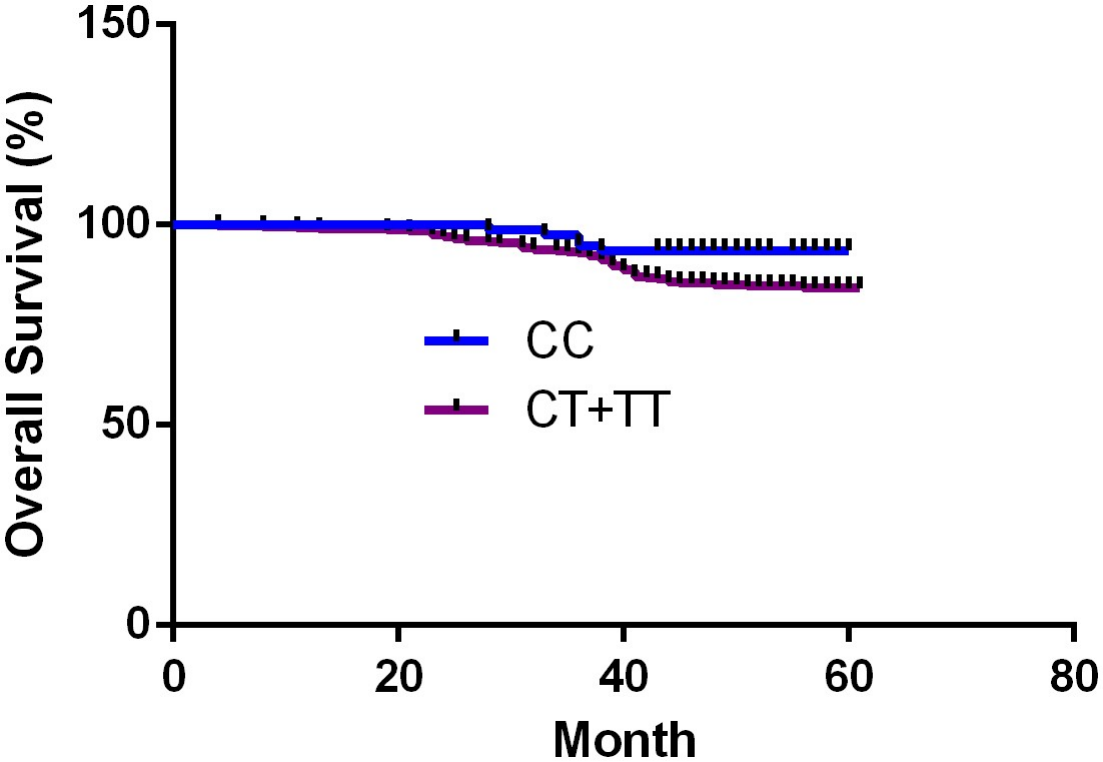
*MTHFR* C677T polymorphism and breast cancer patient survival.

To investigate the association of the *MTHFR* C6*77T* polymorphism and breast cancer risk, we conducted a five-year survival analysis. Our results revealed that BC patients with the *MTHFR* C6*77T* polymorphism (CT+ TT) exhibited a relatively shorter survival time than 677CC carriers (HR, 1.97; 95% Cl, 1.03–3.77; *P* = 0.041) (Fig 1).

## Discussion

In the present study, we investigated whether the *MTHFR* C677T polymorphism is related to the occurrence of different subtypes of BC. We found that the MTHFR C677T polymorphism was substantially associated with luminal B and TNBC susceptibility and exerted a protective effect against the risk of luminal A. Moreover, this study indicated that TNBC patients with the *MTHFR* C677T polymorphism are associated with hypertension, and the same gene polymorphism in luminal B breast cancer patients may be associated with certain clinical characteristics, such as diabetes mellitus and increased BMI. To our knowledge, this study provides evidence that *MTHFR* gene polymorphisms are associated with susceptibility to different BC subtypes and influence relevant clinicopathological features.

Some studies have reported inconsistent data for the significance of *MTHFR* 677C> T, although the *MTHFR* 677T allele was reported to be a potential genetic risk factor for increased BC susceptibility, as determined in a recent meta-analysis [16]. These results suggest that *MTHFR* plays a vital role in the etiology and progression of breast cancer. Furthermore, the *MTHFR* 677C> T polymorphism decreases MTHFR production, which correlates with increased plasma Hcy and decreased plasma FA levels [7]. Many epidemiological studies have demonstrated that dietary folate intake exerts a protective effect against the risk of breast cancer [18, 19]; thus, folate intake insufficiency leads to a high risk of BCs because it may induce disturbances in human chromosomal DNA replication and DNA repair [20].

TNBC (ER^−^/PR^−^/HER2^−^) accounts for approximately 10–20% of all breast cancers. It is regarded as an aggressive disease that affects a young patient population and for which effective targeted therapy is not yet available [4]. As two main luminal-like subclasses, luminal A and luminal B were identified by their estrogen and progesterone status and gene expression profiles [21]. In our study, we found that the *MTHFR* 677C> T polymorphism plays opposite roles in TNBC and luminal A as a high-risk or a protective factor, respectively, related to different estrogen/progesterone statuses, gene expression and mechanisms of breast carcinogenesis. Therefore, the molecular mechanisms of the function between the *MTHFR* 677T allele and TNBC or luminal-like breast cancer subtypes are intriguing and warrant further investigation.

The association between the *MTHFR* 677C> T polymorphism and the clinical feature of BC remains unclear. There is considerable evidence linking low folate status with an increased risk of adverse events, including gestational hypertension [22], and the common C677T polymorphism in the gene encoding the folate-metabolizing enzyme MTHFR is implicated in the development of hypertension [23, 24]. Meanwhile, over half of older patients diagnosed with cancer have chronic conditions, such as hypertension, diabetes mellitus and dyslipidemia [25]. Breast cancer patients with diabetes, in particular, have an increased risk of all-cause mortality [26] and potentially breast cancer recurrence and mortality [27]. Our data showed that the *MTHFR* genotype 677C> T was associated with clinical features, such as diabetes mellitus and BMI in luminal B and hypertension in TNBC, indicating that the *MTHFR* 677C>T polymorphism may be an important risk factor affecting luminal B and TNBC. The potential role of the *MTHFR* 677T allele in BC clinical features requires further verification through animal experiments.

The *MTHFR* 677C> T polymorphism was associated with glucose content in all BC patients, especially in luminal B patients, as shown in Table 4. This finding is consistent with the high diabetes mellitus risk in luminal B patients, indicating that the *MTHFR* 677C> T polymorphism may play a vital role in the balance of glucose in luminal B breast cancer. Regarding the mechanism, some studies have indicated that low-dose folic acid supplementation caused by *MTHFR* genotypes 677C> T reduce serum homocysteine levels but increases glucose concentrations [28]; unfortunately, insulin resistance has been found to be significantly associated with tumor progression in luminal B subtype breast cancer in postmenopausal women [29]. Therefore, we speculate that increased glucose concentrations induced by *MTHFR* genotypes 677C> T may be associated with luminal B tumorigenesis and progression.

Additionally, several limitations should be acknowledged in this study. First, a limited number of *MTHFR C677T* polymorphism samples were studied, and the population of this study was limited to the difference between regions in southwestern China. Second, the sample sizes of both populations were not equivalent, especially for the southwestern Chinese population. Population characteristics were biased, such as the age of the samples or the ratio of males to females. Third, the way the *MTHFR C677T* polymorphism in the *MTHFR* gene affects the development and subtypes of BC is still unclear. Information regarding the *MTHFR C677T* polymorphism and clinical characteristics or other causal relationships in BC patients is lacking and remains to be further investigated. Furthermore, despite its association with the *MTHFR C677T* polymorphism in BC subtypes, the role of rs1801133 in the regulation of folate consumption or glucose deserves further study in the future. The causation of different BC subtypes is the complex multifactorial interplay of genetic and environmental factors, but here, we focused on genetic factors without taking into account certain environmental factors associated with BC subtypes, such as tobacco and alcohol intake and physical activities. Last, although the results of our study provide evidence for the *MTHFR C677T* polymorphism in the *MTHFR* gene as a potential biomarker for use in BC subtype analysis, a prospective study in a larger cohort of ethnically diverse patients is warranted to validate these findings.

Generally, in Han Chinese individuals in southwestern China, the *MTHFR* 677C> T polymorphism may not predispose to each BC subtype, but it may significantly increase the risk of susceptibility in luminal B breast cancer and triple-negative breast cancer.

## Supporting information

S1 File (XLSX)

## References

[1] DeSantis CE, Ma J, Gaudet MM, Newman LA, Miller KD, Goding Sauer A, et al. Breast cancer statistics, 2019. CA Cancer J Clin. 2019;69(6):438–51. Epub 2019/10/03. doi: 10.3322/caac.21583 . 31577379

[2] Waks AG, Winer EP. Breast Cancer Treatment: A Review. JAMA. 2019;321(3):288–300. Epub 2019/01/23. doi: 10.1001/jama.2018.19323 . 30667505

[3] Howlader N, Altekruse SF, Li CI, Chen VW, Clarke CA, Ries LA, et al. US incidence of breast cancer subtypes defined by joint hormone receptor and HER2 status. J Natl Cancer Inst. 2014;106(5). Epub 2014/04/30. doi: 10.1093/jnci/dju055 . 24777111 PMC4580552

[4] Denkert C, Liedtke C, Tutt A, von Minckwitz G. Molecular alterations in triple-negative breast cancer-the road to new treatment strategies. Lancet. 2017;389(10087):2430–42. Epub 2016/12/13. doi: 10.1016/S0140-6736(16)32454-0 . 27939063

[5] Krop I, Ismaila N, Andre F, Bast RC, Barlow W, Collyar DE, et al. Use of Biomarkers to Guide Decisions on Adjuvant Systemic Therapy for Women With Early-Stage Invasive Breast Cancer: American Society of Clinical Oncology Clinical Practice Guideline Focused Update. J Clin Oncol. 2017;35(24):2838–47. Epub 2017/07/12. doi: 10.1200/JCO.2017.74.0472 . 28692382 PMC5846188

[6] Cuorvo LV, Verderio P, Ciniselli CM, Girlando S, Decarli N, Leonardi E, et al. PI3KCA mutation status is of limited prognostic relevance in ER-positive breast cancer patients treated with hormone therapy. Virchows Arch. 2014;464(1):85–93. Epub 2013/11/16. doi: 10.1007/s00428-013-1500-7 . 24233241

[7] Crider KS, Yang TP, Berry RJ, Bailey LB. Folate and DNA methylation: a review of molecular mechanisms and the evidence for folate’s role. Adv Nutr. 2012;3(1):21–38. Epub 2012/02/15. doi: 10.3945/an.111.000992 . 22332098 PMC3262611

[8] Erickson JD. Folic acid and prevention of spina bifida and anencephaly. 10 years after the U.S. Public Health Service recommendation. MMWR Recomm Rep. 2002;51(RR-13):1–3. Epub 2002/10/02. . 12353506

[9] Lamprecht SA, Lipkin M. Chemoprevention of colon cancer by calcium, vitamin D and folate: molecular mechanisms. Nat Rev Cancer. 2003;3(8):601–14. Epub 2003/08/02. doi: 10.1038/nrc1144 . 12894248

[10] Chen S, Rong L, Lei Q, Cao PX, Qin SY, Zheng DW, et al. A surface charge-switchable and folate modified system for co-delivery of proapoptosis peptide and p53 plasmid in cancer therapy. Biomaterials. 2016;77:149–63. Epub 2015/11/26. doi: 10.1016/j.biomaterials.2015.11.013 . 26599622

[11] Christensen BC, Kelsey KT, Zheng S, Houseman EA, Marsit CJ, Wrensch MR, et al. Breast cancer DNA methylation profiles are associated with tumor size and alcohol and folate intake. PLoS Genet. 2010;6(7):e1001043. Epub 2010/08/06. doi: 10.1371/journal.pgen.1001043 . 20686660 PMC2912395

[12] Friso S, Choi SW, Girelli D, Mason JB, Dolnikowski GG, Bagley PJ, et al. A common mutation in the 5,10-methylenetetrahydrofolate reductase gene affects genomic DNA methylation through an interaction with folate status. Proc Natl Acad Sci U S A. 2002;99(8):5606–11. Epub 2002/04/04. doi: 10.1073/pnas.062066299 . 11929966 PMC122817

[13] Ferrari A, Torrezan GT, Carraro DM, Aguiar S Junior. Association of Folate and Vitamins Involved in the 1-Carbon Cycle with Polymorphisms in the Methylenetetrahydrofolate Reductase Gene (MTHFR) and Global DNA Methylation in Patients with Colorectal Cancer. Nutrients. 2019;11(6). Epub 2019/06/21. doi: 10.3390/nu11061368 . 31216671 PMC6627304

[14] Castiglia P, Sanna V, Azara A, De Miglio MR, Murgia L, Pira G, et al. Methylenetetrahydrofolate reductase (MTHFR) C677T and A1298C polymorphisms in breast cancer: a Sardinian preliminary case-control study. Int J Med Sci. 2019;16(8):1089–95. Epub 2019/09/17. doi: 10.7150/ijms.32162 . 31523170 PMC6743281

[15] Zhang XF, Liu T, Li Y, Li S. Association between MTHFR 677C/T and 1298A/C gene polymorphisms and breast cancer risk. Genet Mol Res. 2015;14(4):16425–30. Epub 2015/12/15. doi: 10.4238/2015.December.9.12 . 26662439

[16] Meneses-Sanchez P, Garcia-Hernandez SC, Porchia LM, Perez-Fuentes R, Torres-Rasgado E, Del Angel Soto A, et al. C677T and A1298C methylenetetrahydrofolate reductase polymorphisms and breast cancer susceptibility among Latinos: a meta-analysis. Breast Cancer. 2019;26(5):602–11. Epub 2019/03/17. doi: 10.1007/s12282-019-00961-8 . 30877449

[17] Schoemaker MJ, Nichols HB, Wright LB, Brook MN, Jones ME, O’Brien KM, et al. Adult weight change and premenopausal breast cancer risk: A prospective pooled analysis of data from 628,463 women. Int J Cancer. 2020. Epub 2020/02/06. doi: 10.1002/ijc.32892 . 32012248 PMC7365745

[18] Castillo LC, Tur JA, Uauy R. [Folate and breast cancer risk: a systematic review]. Rev Med Chil. 2012;140(2):251–60. Epub 2012/06/29. doi: 10.4067/S0034-98872012000200016 . 22739957

[19] Chen P, Li C, Li X, Li J, Chu R, Wang H. Higher dietary folate intake reduces the breast cancer risk: a systematic review and meta-analysis. Br J Cancer. 2014;110(9):2327–38. Epub 2014/03/29. doi: 10.1038/bjc.2014.155 . 24667649 PMC4007237

[20] Kawakita D, Lee YA, Gren LH, Buys SS, La Vecchia C, Hashibe M. The impact of folate intake on the risk of head and neck cancer in the prostate, lung, colorectal, and ovarian cancer screening trial (PLCO) cohort. Br J Cancer. 2018;118(2):299–306. Epub 2017/11/22. doi: 10.1038/bjc.2017.383 . 29161239 PMC5785740

[21] Sorlie T, Perou CM, Tibshirani R, Aas T, Geisler S, Johnsen H, et al. Gene expression patterns of breast carcinomas distinguish tumor subclasses with clinical implications. Proc Natl Acad Sci U S A. 2001;98(19):10869–74. Epub 2001/09/13. doi: 10.1073/pnas.191367098 . 11553815 PMC58566

[22] De Ocampo MPG, Araneta MRG, Macera CA, Alcaraz JE, Moore TR, Chambers CD. Folic acid supplement use and the risk of gestational hypertension and preeclampsia. Women Birth. 2018;31(2):e77–e83. Epub 2017/09/06. doi: 10.1016/j.wombi.2017.08.128 . 28870524

[23] McNulty H, Strain JJ, Hughes CF, Ward M. Riboflavin, MTHFR genotype and blood pressure: A personalized approach to prevention and treatment of hypertension. Mol Aspects Med. 2017;53:2–9. Epub 2016/10/11. doi: 10.1016/j.mam.2016.10.002 . 27720779

[24] McNulty H, Strain JJ, Hughes CF, Pentieva K, Ward M. Evidence of a Role for One-Carbon Metabolism in Blood Pressure: Can B Vitamin Intervention Address the Genetic Risk of Hypertension Owing to a Common Folate Polymorphism? Curr Dev Nutr. 2020;4(1):nzz102. Epub 2020/01/21. doi: 10.1093/cdn/nzz102 . 31956853 PMC6955829

[25] Ritchie CS, Kvale E, Fisch MJ. Multimorbidity: an issue of growing importance for oncologists. J Oncol Pract. 2011;7(6):371–4. Epub 2012/03/02. doi: 10.1200/JOP.2011.000460 . 22379419 PMC3219463

[26] Barone BB, Yeh HC, Snyder CF, Peairs KS, Stein KB, Derr RL, et al. Long-term all-cause mortality in cancer patients with preexisting diabetes mellitus: a systematic review and meta-analysis. JAMA. 2008;300(23):2754–64. Epub 2008/12/18. doi: 10.1001/jama.2008.824 . 19088353 PMC3093051

[27] Calip GS, Yu O, Hoskins KF, Boudreau DM. Associations between diabetes medication use and risk of second breast cancer events and mortality. Cancer Causes Control. 2015;26(8):1065–77. Epub 2015/05/10. doi: 10.1007/s10552-015-0599-z . 25956271 PMC4501774

[28] Chmurzynska A, Malinowska AM, Twardowska-Rajewska J, Gawecki J. Elderly women: Homocysteine reduction by short-term folic acid supplementation resulting in increased glucose concentrations and affecting lipid metabolism (C677T MTHFR polymorphism). Nutrition. 2013;29(6):841–4. doi: 10.1016/j.nut.2012.09.015 23298970

[29] Nam S, Park S, Park HS, Kim S, Kim JY, Kim SI. Association Between Insulin Resistance and Luminal B Subtype Breast Cancer in Postmenopausal Women. Medicine (Baltimore). 2016;95(9):e2825–e. doi: 10.1097/MD.0000000000002825 . 26945364 PMC4782848

